# A Dual Valorization Strategy of Barley Straw for the Development of High-Performance Bio-Based Polyurethane Foams

**DOI:** 10.3390/polym17233142

**Published:** 2025-11-26

**Authors:** Marina Rodríguez-Aranda, Esther Rincón, María Pinillos, Pablo E. Romero, Luis Serrano

**Affiliations:** 1 BioPrEn RNM-940 Research Group, Inorganic Chemistry and Chemical Engineering Department, Universidad de Córdoba, Marie Curie Building (C-3), Ctra. Nnal. Km. 396, 14014 Córdoba, Spain; q92roarm@uco.es (M.R.-A.);; 2 Faculty of Science, Instituto Químico Para la Energía y el Medioambiente (IQUEMA), Universidad de Córdoba, Marie Curie Building (C-3), Ctra. Nnal. Km. 396, 14014 Córdoba, Spain; 3 Mechanical Engineering Department, Universidad de Córdoba, Leonardo da Vinci Building, Ctra. Nnal. Km. 396, 14014 Córdoba, Spain

**Keywords:** polyurethane foams, lignin, barley straw, lignin-containing cellulose nanofibers, biorefinery

## Abstract

This study presents a complete and zero-waste valorization strategy for barley straw through the synthesis of bio-polyols and the concurrent utilization of its cellulose fraction as lignin-containing cellulose nanofibers (LCNF) for the development of bio-based polyurethane (PU) foams. Two types of bio-polyols were prepared: one derived from lignin isolated via biomass fractionation, named lignin bio-polyol (LBP), and another obtained directly from unfractionated barley straw, called straw bio-polyol (SBP), thereby incorporating all lignocellulosic constituents into a single reactive matrix. LCNF, produced from the same feedstock, was incorporated at different loadings to achieve full biomass utilization and reinforce the polyurethane foam structure. Foams prepared with LBP exhibited lower density and a more homogeneous structure, whereas those synthesized with SBP developed a stiffer, more crosslinked network. The incorporation of LCNF generally increased apparent density and mechanical performance, with optimal reinforcement at 3 wt.% in foams with SBP and 5 wt.% in LBP foams, corresponding to a 62.5 and 121% enhancement in compressive strength relative to their respective control foams. Moreover, the use of barley straw bio-polyol shifted some thermal degradation peaks toward higher temperatures, evidencing improved thermal resistance. Overall, this dual-route strategy provides a sustainable and versatile methodology for the comprehensive valorization of lignocellulosic biomass, enabling a systematic understanding of the role of each structural component in polyurethane foam synthesis. The resulting materials offer a renewable, low-impact pathway toward high-performance polymeric materials.

## 1. Introduction

In the latter half of the 20th century, the plastic industry experienced exponential growth, a trend expected to persist in the coming decades. In 2023, global plastic production reached 413.8 Mt [1]. This sustained expansion has intensified environmental concerns, driven by the accelerated generation of plastic waste and its propensity to accumulate in landfills or enter marine ecosystems [2]. Among all plastics, polyurethanes represent approximately 5.3% of total production, largely owing to their remarkable versatility, which enables applications ranging from construction to biomedical engineering [3]. Within this family, foams dominate the market, representing roughly 65% of overall PU output [4]. Their combination of low thermal conductivity, mechanical robustness, and excellent acoustic insulation renders them indispensable materials for energy-efficient construction and advanced engineering applications [5].

Since their development in the 1930s, PU materials have been synthesized predominantly from petroleum-derived polyols and isocyanates. Although these components are essential for urethane bond formation, their reliance on finite fossil resources and their associated toxicity pose significant environmental and health challenges [6]. As a result, the development of sustainable synthetic routes capable of reducing fossil dependence and mitigating pollution during synthesis has become a critical research priority. In this context, biorefinery strategies offer an attractive framework for the valorization of lignocellulosic biomass into renewable chemicals and materials, introducing environmentally benign synthetic pathways and helping stabilize the price volatility of petroleum-derived materials [7].

Agricultural residues are generated in large quantities annually and typically contain 30–60% cellulose, 20–40% hemicellulose, and 15–25% lignin [8]. However, despite their intrinsic value, these residues are often improperly managed, leading to environmental burdens such as open field burning or uncontrolled decomposition. Moreover, given their abundance, low cost and heterogeneous composition, lignocellulosic biomass represents an ideal renewable feedstock for producing high-value products, representing a key step toward improving resource efficiency and advancing the circular economy. Accordingly, the chemical transformation of lignocellulosic biomass into renewable intermediates for composites, epoxies and polyurethanes has attracted scientific attention [9,10,11,12,13].

Among the major structural components of biomass, lignin remains one of the most underutilized. Traditionally generated as a by-product of the pulp and paper industry, lignin is mostly burned as low-grade fuel, limiting its economic potential [14]. Structurally, lignin is a complex three-dimensional aromatic polymer composed of p-coumaryl, coniferyl and sinapyl alcohol units. Its abundance of hydroxyl, carboxyl, carbonyl and methoxy groups provides high chemical reactivity and tunability [15,16]. This rich functionality positions lignin as a promising feedstock for thermochemical conversion processes such as pyrolysis, gasification and liquefaction [17].

Among these, liquefaction is particularly attractive, as it enables the depolymerization of lignin and its conversion into bio-polyols using a polyhydric alcohol as solvent, producing high molecular weight polymers with favorable characteristics for polyurethane production, such as high fluidity and abundant hydroxyl content [18]. Furthermore, lignin’s non-crystalline, irregular structure has been shown to enhance key aspects of polyurethane foams, including mechanical performance, thermal stability and component miscibility [19]. While lignin-based bio-polyols can partially replace petroleum-derived polyols, achieving appropriate soft-to-hard segment balance in PU foams requires blending with compatible chain extenders [20]. In this regard, non-edible, hydroxyl-rich vegetable oils, such as castor oil, serve as sustainable chain extenders that integrate seamlessly into the polymer network, enabling precise control over foam flexibility and overall material performance [21,22,23].

However, to move closer to complete biomass valorization and advance toward a truly zero-waste process, the cellulose fraction must also be considered. Its transformation to the nanoscale produces cellulose nanofibers (CNF) or lignin-containing cellulose nanofibers (LCNF), which exhibit low density, high surface area and abundant functionality, enabling their effective integration into polymer matrices as fillers or reinforcing agents [24,25,26]. In contrast to CNF, whose production requires energy-intensive bleaching to remove lignin, LCNF can be obtained without this step, resulting in both economic and environmental advantages. Beyond acting as structural reinforcement, LCNF incorporation in PU foams can also enhance mechanical and thermal performance. Moreover, its lignin content provides unique structural and interfacial advantages such as hydrophobicity, which could reduce the surface polarity of the nanofibers and improve their compatibility with aromatic and hydrophobic segments of the polyurethane network while promoting full biomass utilization and reducing dependency on petrochemical inputs [27,28]. However, the potential of LCNF as a structural reinforcement in bio-based polyurethane foams remains largely unexplored, highlighting an important opportunity for exploration.

The present study introduces a dual-biorefinery approach enabling the full valorization of barley straw while producing high-performance bio-based PU foams, thereby offering a sustainable alternative to petroleum-derived materials and contributing to the mitigation of agricultural residue management challenges. First, a lignin bio-polyol was synthesized via liquefaction as a sustainable partial replacement for petroleum-derived polyols, combined with freeze-dried LCNF at different loadings to enhance structural integrity while promoting a zero-waste approach. Second, to prevent energy-intensive fractionation required for lignin isolation, a bio-polyol was obtained via direct liquefaction of whole barley straw, also reinforced with LCNF, allowing for a systematic comparison between formulations. This integrated approach not only provides a comprehensive evaluation of the influence of bio-polyol composition on foam performance but also establishes a sustainable, low-impact route for producing advanced bio-based polyurethane materials.

## 2. Materials and Methods

### 2.1. Materials

Barley straw used as raw material was supplied by a local farmer. It was ground using an SM 200 Retsch hammer mill and sieved to obtain a 4–6 cm fraction. The raw material was characterized according to standard TAPPI methods [29], yielding the following average composition: 36.5% cellulose, 25.6% hemicelluloses, 13.1% lignin, 6.9% ash and 11.6% alcohol-extractable compounds. The reagents used included NaOH (98%, Panreac Applied Chem, Barcelona, Spain), pyridine (Labkem, Mataró, Spain), acetic anhydride (99%, Labkem), 1,4-dioxane (99.5%, PanReac) and KOH (85%, PanReac). The liquefaction reaction was conducted using glycerol (99%, Labkem) and H_2_SO_4_ (96%, Panreac). For the synthesis of LCNF, the following reagents were used: TEMPO (98%, Sigma-Aldrich, St. Louis, MO, USA), NaClO (10% *w*/*v*, Panreac Applied Chem) and NaBr (Honeywell, Seelze, Germany). The production of polyurethane foam involved castor oil (Sigma-Aldrich), dibutyltin dilaurate (95%, Sigma-Aldrich), silicon oil (Sigma-Aldrich) and tolylene 2,4-diisocyanate (80%, Sigma-Aldrich).

### 2.2. Biomass Fractionation

Barley straw was treated under reaction conditions optimized by Espinosa et al. [30] for maximizing lignin removal while preserving cellulose integrity. A pressurized batch reactor equipped with an outer jacket was used at 100 °C, 7% NaOH, and a liquid-to-solid ratio of 10:1 for 150 min while maintaining constant agitation. The resulting solid fraction was then processed in a pulp disintegrator for 30 min and passed through a Sprout-Bauer beater. Subsequently, the content was sieved through a 0.14 mm mesh, yielding cellulose pulp. The black liquor generated during the pulping process was acidified with H_2_SO_4_ until pH 2; further, a selective centrifugation at 3500 r.p.m. was carried out. Then, the solid was dried at 40 °C for the recovery of the lignin fraction.

### 2.3. Liquefaction

Bio-polyols were synthesized via acid-catalyzed liquefaction of either isolated lignin (producing LBP) or untreated barley straw (producing SBP). In a typical procedure, 20 g of feedstock were placed in a glass reactor, together with 100 mL of glycerol and 3 g of H_2_SO_4_. A mechanical stirrer, a thermocouple and a condenser were integrated into the reactor. The reaction mixture was heated to 180 °C for 1 h, a procedure previously optimized by Rincón et al. [31]. After liquefaction, the viscous product was filtered to remove the unreacted residues. The recovered solid was washed with acetone and oven-dried at 100 °C for 24 h to calculate the reaction yield using Equation (1).

(1) $$\mathrm{Liquefaction}\;\mathrm{yield}=\left[1-\frac{M}{M_{0}}\right]\times 100$$

where M is the dry weight of unreacted reagents and M_0_ is the weight of lignin or barley straw initially added to the reactor.

### 2.4. Bio-Polyol Characterization

The pH of the bio-polyols was determined using a pH meter (pH GLP21, Crison, Barcelona, Spain) after dispersing 1 g of sample in 50 mL of distilled water. The density was measured by weighing between 1 and 2 mL of the polymer. The viscosity was evaluated at 25 °C with a Brookfield Viscometer (Fungilab, Barcelona, Spain) at 70 and 85 r.p.m. for the barley straw bio-polyol and lignin bio-polyol, respectively. The functionality of the bio-polyols was assessed through their hydroxyl number (I_OH_) and acid number. The I_OH_ was determined by esterifying 1.4 g of bio-polyol with 20 mL of phthalation reagent (200 mL of pyridine along with 25.4 mL of acetic anhydride) at 98 °C for 2 h. Subsequently, 100 mL of distilled water and a 1% phenolphthalein indicator solution in pyridine were added, and the mixture was titrated with 0.5 N NaOH. The acid number was measured by titrating a 0.4 g sample dissolved in 50 mL of dioxane–water (4:1) solution with 0.1 M KOH. The molecular weight distribution was analyzed by size-exclusion chromatography (SEC) using a PL-GPC 50 system (Agilent Technologies, Santa Clara, CA, USA) equipped with a refractive index detector and operating at 50 °C. KD-G 4A and KD-806M columns (Shodex, Tokyo, Japan) were employed. Samples were dissolved in DMF at a concentration of 2 mg/mL, filtered and injected (100 μL) at a flow rate of 1 mL/min. Bio-polyols were also analyzed by attenuated total reflectance Fourier transform infrared spectroscopy (ATR-FTIR) in a Perkin-Elmer Spectrum Two, collecting 20 scans between 4000 and 400 cm^−1^ with a resolution of 4 cm^−1^.

### 2.5. LCNF Production

Lignin-containing cellulose nanofibers were produced by a mechano-chemical methodology that included a pretreatment based on the TEMPO-mediated reaction. This procedure involves the oxidation of cellulose pulp with 2,2,6,6-tetramethylpiperidinyl-1-oxyl (TEMPO) as a catalyst. Specifically, 10 g of cellulose pulp were suspended in water containing 0.16 g of TEMPO and 1 g of sodium bromide. Then, 5 mmol of NaClO per gram of pulp (30 mL) was added to the reaction mixture. After the complete addition, 0.5 M NaOH was gradually introduced to maintain the pH slightly above 10. Once the pH remained stable for 2 h, the reaction was quenched with ethanol. The oxidized pulp was subsequently fibrillated using a high-pressure homogenizer (PANDA 2000, Gea Niro Soavi, Parma, Italy) operating with a 1% fiber suspension. The resulting LCNF was freeze-dried and milled prior to its incorporation into the polyurethane foams.

### 2.6. Quality Index of LCNF

The LCNF quality was evaluated following the method proposed by Desmaisons et al. [32], which applies a simplified Quality Index (Q.I.*) procedure based on four key parameters. The nanosize fraction, turbidity, Young’s modulus, and macroscopic size were determined in triplicate, and the corresponding mean values and standard deviation were reported. These parameters were then introduced into Equation (2) to determine the Q.I.*

Q.I.* = 0.30·x_1_ + (−0.03·x_2_) − 0.071·x_3_^2^ +2.54·x_3_ − 5.35·ln(x_7_) + 59.9 (2)

where x_1_ is the nanosize fraction (%), x_2_ is turbidity (NTU), x_3_ is Young’s modulus (GPa) and x_7_ is macroscopic size (µm^2^).

### 2.7. PU Foams Synthesis

The foams were synthesized through a free-rise process. The formulations were prepared in a plastic cup by sequentially incorporating castor oil as a bio-based chain extender, the selected bio-polyol (either LBP or SBP), dibutyltin dilaurate as a catalyst, silicone oil as a surfactant, distilled water as a chemical blowing agent and freeze-dried LCNF at different loadings (1, 3, 5 and 7 wt. %). The mixture was homogenized using a mechanical stirrer at 1000 r.p.m. for 1 min. Subsequently, TDI was added, and the system was stirred under identical conditions. Blending parameters were selected to ensure uniform dispersion and effective integration of the freeze-dried LCNF. Bio-polyol: castor oil ratio was optimized considering earlier findings related to successful foaming [33]. Among the two series of samples (LBP or SBP foams), those displaying the most balanced combination of structural integrity and mechanical performance under open-mold conditions were reproduced in closed mold, as they represent the most suitable candidates for assessing the effect of confined expansion (typical of industrial processing) on the final foam structure and properties [34]. Detailed formulations are provided in Table 1.

Isocyanate/hydroxyl molar ratio was calculated using Equation (3):

(3) $$R_{NCO/OH}=\frac{M_{NCO}\cdot W_{NCO}}{\left[M_{bio-polyol}\cdot W_{bio-polyol}\;+\;M_{castoroil}\cdot W_{castoroil}\;+\;M_{water}\cdot W_{water}\right]}$$

where M is the number of moles of the corresponding reagent per gram of compound, and W is the weight added to the reaction mixture.

### 2.8. PU Foams Characterization

According to ASTM D1622-23 [35], the apparent density of the PU foams was determined. Cubic specimens with 3.5 cm sides were cut from the central region of each sample and weighed. The apparent density was calculated based on the average of three replicates.

Mechanical behavior was evaluated using a Lloyd LF Plus (Lloyd Instruments Ltd., Fareham, Hampshire, UK). During the test, each foam was placed between two parallel plates moving towards each other at a speed of 0.21 cm/min. The applied force was stopped when the strain reached 10% of the initial specimen height.

Thermal stability was analyzed by thermogravimetric analysis (TGA) using a Mettler-Toledo TGA/DSC (Mettler-Toledo, Barcelona, Spain) system. The samples were heated from 50 °C to 800 °C at a rate of 10 °C/min under a constant nitrogen flow of 50 mL/min. For differential scanning calorimetry (DSC) analysis, all samples were purged with nitrogen at a flow rate of 40 mL/min. The first heating scan was carried out from 25 to 100 °C to eliminate thermal memory. Subsequently, a cooling cycle was carried out down to 50, followed by a heating scan up to 250 °C.

The cell morphology of the synthesized polyurethane foams was examined by field emission scanning electron microscopy (JEOL JSM 7800F, JEOL Ltd. Tokyo, Japan) operated at 5 kV and 30× magnification. Samples were cut into 1 × 1 cm squares, placed on an SEM mount using carbon tape and sputter-coated with a 50 Å gold layer. The average cell size and average cell wall thickness were determined using ImageJ software version 1.54g by analyzing ten images per sample.

FTIR-ATR was carried out in polyurethane samples in a Perkin-Elmer Spectrum Two (Perkin Elmer, Waltham, MA, USA) running 20 scans with a resolution of 4 cm^−1^ between 4000 and 400 cm^−1^.

## 3. Results and Discussion

### 3.1. Liquefaction and Bio-Polyol Characterization

Liquefaction of lignin and barley straw yielded 98.96% and 99.43%, respectively, confirming the high efficiency of the process. Such elevated yields are attributed to the reaction temperature (180 °C), which promotes both biomass depolymerization and secondary recondensation phenomena. The negligible amount of residual solid indicates an almost complete conversion of the lignocellulosic feedstock. These results are consistent with conversion efficiencies previously reported for similar feedstocks, as Kraft lignin [36], palm kernel [37] or rape straw [38].

Given the intended application of the bio-polyols in polyurethane foams synthesis, their physicochemical properties were evaluated to ensure compatibility with foam-forming reactions. Among the most critical parameters are the hydroxyl number (I_OH_) and acid number, which govern the crosslinking potential. For rigid and semi-rigid polyurethane foams, suitable polyols typically exhibit I_OH_ values between 300 and 800 mg KOH/g [39]. The bio-polyols obtained in this work displayed I_OH_ values of 710.8 and 625.7 mg KOH/g for lignin and barley straw bio-polyol, respectively, well within the optimal range for polyurethane foam production. However, a high acid number can adversely affect polymerization kinetics by neutralizing tertiary amine catalysts [40,41]. The acid number was significantly higher for the lignin bio-polyol (49.9 mg KOH/g) than for the barley straw bio-polyol (22.7 mg KOH/g), reflecting the partial oxidation of the different lignocellulosic fragments during liquefaction. Despite the difference, both values remain within acceptable limits for foam synthesis.

From a rheological standpoint, viscosity plays a key role in ensuring homogeneous dispersion and complete reaction between precursors. To maintain sufficient mixing and polymer chain mobility, viscosity should remain below 300 Pa·s [42]. As summarized in Table 2, both bio-polyols met this requirement, thereby confirming their suitability for polyurethane foam preparation.

The FTIR spectra of both bio-polyols (Figure S1), along with a detailed description of their principal characteristic peaks, are included in the Supplementary Material.

### 3.2. LCNF Quality Index

It is well established that the distinctive properties of LCNF are closely linked to the fraction of nanoscale components present in the suspensions. In this study, the nanosize particle content reached 94.87%, confirming that the applied treatment was effective for the intended nanofibrillation. Indirectly, this parameter was also assessed through turbidity measurements, which quantify light scattering caused by particle aggregation. For highly nanostructured suspensions, turbidity values tend to approach zero; thus, the obtained value (63.33 NTU) is consistent with the high nanoscale fraction, reinforcing the reliability of the analysis. The mechanical response of the LCNF network (evaluated indirectly through the Young’s modulus of LCNF nanopaper) provides additional insights into fiber morphology and cellulose interactions. The measured Young’s modulus (9.30 GPa) was slightly lower than those reported in similar studies, suggesting a moderately reduced network connectivity and bonding density [43]. This observation agrees with the macroscopic area of residual fibers in suspension (9.4 µm^2^), which reflects an adequate but not complete delamination of the original cell wall structure [44]. The Quality Index integrates information on fiber dispersion, inter-fiber interactions and size distribution, enabling a comparative evaluation of different LCNF suspensions. In this work, the simplified quality index reached 93.4%, indicating a highly homogenous dispersion with minimal aggregation, key attributes for maximizing surface area, reactivity and LCNF performance. Although originally developed for bleached cellulose material, this index has recently been validated as an approximate yet reliable tool for assessing LCNF [45]. Further details are summarized in Table 3.

### 3.3. Polyurethane Foams Synthesis

Polyurethane foams were successfully synthesized incorporating over 63 wt.% of renewable reagents. These formulations align with a zero-waste process, as both aromatic and cellulosic fractions of the biomass are directly integrated into the different foam systems, ensuring that all major components of the feedstock contribute to material functionality. This approach not only underscores the relevance of each fraction within the final polymer network but also enables a clearer understanding of their individual roles and their synergistic effects.

A higher substitution degree of castor oil was achieved with the barley straw bio-polyol (50:50 ratio), compared to the lignin bio-polyol (40:60). This behavior can be ascribed to the aromatic and three-dimensional structure of the lignin-based bio-polyol, where steric hindrance partially limits the interaction between hydroxyl groups and the isocyanate. The use of tolylene diisocyanate (TDI), an isomeric mixture of 2,4- and 2,6-TDI, may accentuate this effect due to its asymmetric structure, which can hinder or reduce the formation of hard segments [46]. Consequently, formulations containing components with predominantly aromatic moieties are expected to yield foams with higher flexibility and reduced density [47].

Given that water plays a critical role in gas formation during PU production, its availability in the reactive mixture must be precisely controlled. To avoid uncontrolled moisture interference, the LCNF hydrogel was therefore lyophilized prior to incorporation. As LCNF was expected to act as a source of hydroxyl groups, its influence was evaluated at a fixed NCO/OH ratio. To determine the saturation threshold of the polymer matrix with these functional groups, foams containing different LCNF loadings were synthesized. It is worth noting that foaming was not achieved at 7 wt.% LCNF, likely because the high volume of freeze-dried LCNF added, which could hinder the direct contact between isocyanate and water, or likely due to the saturation of isocyanate with an excessive amount of hydroxyl groups, which could suppress the blowing reaction necessary for foam expansion.

### 3.4. Polyurethane Foams Characterization

#### 3.4.1. Physicochemical and Morphological Characterization of PU Foams

**Figure 1:**
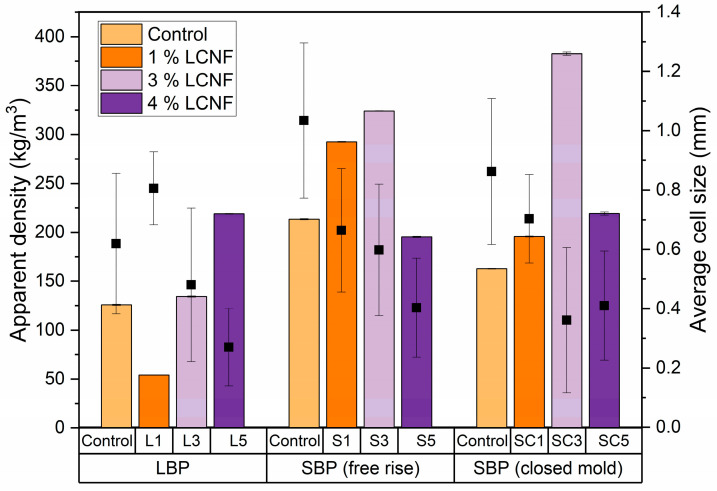
Apparent density and average cell size of foams. (Apparent density represented by bar charts and average cell size indicated by data points).

**Figure 2:**
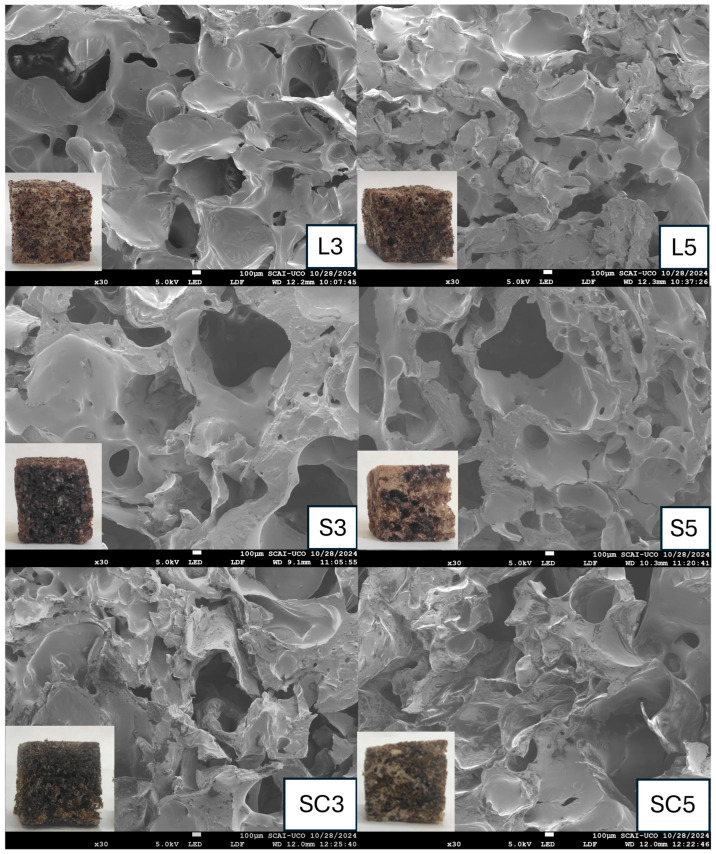
SEM images of PU foams.

The apparent density values and average cell size of PU foams are shown in Figure 1. Overall, the incorporation of LCNF led to an increase in apparent density, primarily associated with the significant reduction in cell size observed in SEM images (Figure 2). The densest foams within each formulation group exhibited density increases of 74% (L5), 51% (S3) and 135% (SC3) compared with the corresponding control foams. This behavior can be attributed to additional linkages between the primary hydroxyl groups of LCNF and the isocyanate groups of TDI [48]. In this way, LCNF would compete with the bio-polyols for isocyanate reactive sites, leading to a more crosslinked and compact polymeric network and, consequently, a smaller cell size, consistent with previous reports [28,49]. The average decrease in cell size of 5% LCNF foams with respect to control foams was 57%, which can be explained by the role of LCNF as nucleating agent, which could provide energetically favorable interfaces for heterogeneous bubble nucleation due to its high specific surface area and could produce a decrease in bubble coalescence, leading to a higher number of smaller cells [28]. However, the reduction in cell size did not follow a strictly proportional trend with LCNF loading in any of the formulations. These results are in line with literature observations showing that cell size and density in PU foams depend not only on the NCO/OH ratio, but also on the specific formulation and filler dispersion, rather than responding linearly to the filler concentration [50]. Simultaneously with the decrease in cell size, LCNF incorporation produced an increase in average cell wall thickness of 78%. It could be attributable to the partial immobilization of polymer chains at the nanofiber surface that could reinforce the cell wall during expansion, preventing excessive thinning and contributing to the formation of a denser cellular skeleton. Further details about apparent density, cell size and cell wall thickness can be found in Table S1 and Table S2, respectively (see Supplementary Material).

When comparing free-rise foams, those prepared with LBP generally exhibited lower densities than those produced with SBP. As discussed earlier, this trend can be explained by the steric hindrance introduced by the aromatic moieties (from lignin monomers and TDI) [46]. Although the LBP presented a higher hydroxyl number, many of these groups could be sterically inaccessible for reaction, reducing effective polymerization. In contrast, the SBP, which retains all lignocellulosic structural components (cellulose, hemicelluloses, and lignin), facilitated a more effective polymer network formation due to its lower aromatic character.

One exception to this general trend was observed. The L1 sample exhibited lower density than its respective control, showing a more homogeneous and sponge-like appearance. Similar behavior was reported by Ghasemi et al. [51], who observed that the addition of 0.8 wt.% CNF led to an increase in cell size relative to unfilled foams. Likewise, in S5 and SC5, the density decreased compared with S3 and SC3, respectively. This effect can be attributed to the different hydroxyl group availability of the bio-polyols. In the case of SBP, the higher content of accessible hydroxyl groups suggests that LCNF may lead to matrix saturation. Conversely, for LBP, where hydroxyl groups are more sterically hindered, the progressive incorporation of LCNF compensates for this limitation, providing additional reactive sites, which has also been noted by Huang et al. [49] at comparable reinforcement levels. Finally, foams produced under controlled expansion conditions displayed significantly lower density in some cases than their open-mold counterparts, with evident cell deformation and wall rupture. However, these morphological differences did not translate into a statistically significant variation in the average cell size, indicating that confinement primarily influenced the cell anisotropy rather than differences in porosity.

**Figure 3:**
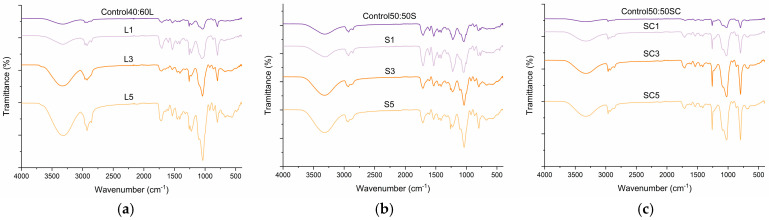
(**a**) FTIR spectra of lignin bio-polyol foams; (**b**) FTIR spectra of straw bio-polyol foams; (**c**) FTIR spectra of straw bio-polyol foams in a closed mold.

Additionally, the FTIR spectra of the polyurethane foams (Figure 3) displayed the characteristic vibrational features of urethane-based polymer networks, along with systematic variations associated with the incorporation of LCNF. All formulations exhibited a broad absorption band centered at 3300 cm^−1^, corresponding to N-H stretching of urethane linkages, and a well-defined band near 1725 cm^−1^, assigned to the C=O stretching of urethane groups. Both bands increased in intensity upon LCNF addition, indicating a higher proportion of urethane linkages formed through reactions between isocyanate groups and the hydroxyl functionalities supplied by LCNF, confirming the initial hypothesis. This behavior is consistent with the development of a more extensively crosslinked polymer network in reinforced foams. Moreover, this trend is not observed between S3-S5 and SC3-SC5 samples, supporting the results reported in the previous analysis. Additionally, the sharp band observed at approximately 1100 cm^−1^, attributed to C-O-C stretching vibrations of ether linkages of bio-polyol, reflects the final presence of bio-polyol residual content in the final polymer matrix, which increases with the addition of LCNF. Overall, the spectral changes confirm the role of LCNF as a reactive component, increasing the density of urethane linkages and enhancing matrix cohesion [27].

#### 3.4.2. Mechanical Properties

**Figure 4:**
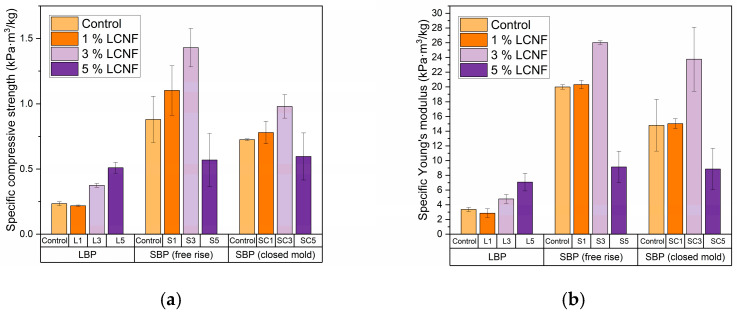
(**a**) Specific compressive strength for PU foams; (**b**) Young’s modulus values of PU foams.

As illustrated in Figure 4a,b, both compressive strength and Young’s modulus increased upon the incorporation of LCNF, confirming its effective reinforcing role within the polyurethane matrix. It could be attributable to the high-aspect-ratio of LCNF, which could promote more efficient stress transfer across the polymer-filler interface. Their geometry could facilitate interlocking with the polyurethane network, increasing the reinforcement efficiency even at low loadings. The most pronounced enhancement was observed in foams derived from LBP, where compressive strength and Young’s modulus increased by 121% and 111%, respectively, at the highest reinforcement level (5 wt.%) relative to the unfilled control. This reference foam displayed mechanical performance consistent with literature data [52], which validates the reliability of the experimental approach. Its comparatively lower compressive strength compared with SBP foams is attributed to a less crosslinked polymer network, given that lignin-derived aromatic monomers were the structural units available for urethane formation. Nevertheless, the combination of moderate stiffness and high sponginess suggests potential applicability in automotive interiors or cushioning products where resilience, energy dissipation, and long-term durability are key requirements.

In contrast, the foams prepared with SBP exhibited significantly higher absolute values of compressive strength and Young’s modulus, achieving maximum performance in the open mold formulation S3 (1.43 kPa·m^3^/kg and 26 kPa·m^3^/kg, respectively) [53]. Although the relative improvement with LCNF addition was less pronounced (62.5% in compressive strength and 30.6% in modulus), these results highlight the enhanced structural performance of bio-polyols derived from whole lignocellulosic biomass. However, at the highest LCNF loading (S5), a reduction in compressive strength and Young’s modulus was detected, consistent with previous observations that confirm that a high proportion of filler can decrease the mechanical performance of the material due to the lack of uniformity in filler dispersion [54,55]. Overall, foams synthesized with SBP exhibit mechanical requirements aligned with structural applications within the construction sector.

Although closed-mold foams followed a similar trend to their free-rise counterparts, a general reduction in mechanical performance was observed. This behavior may arise from the increased internal stresses during foam growth that promote cell deformation, ultimately resulting in a less uniform cellular structure. Such microstructural imperfections hinder the efficient distribution of applied loads and compromise the overall strength of the material. Overall, the confined foaming conditions inherent to closed mold processing restrict uniform cell growth and promote structural anisotropy, which collectively account for the lower mechanical response observed.

In this way, by evaluating the resulting foams, this strategy provides a systematic screening of structure-property relationships, facilitating the selection of the most suitable components for specific performance requirements and targeted applications.

#### 3.4.3. Thermal Properties

**Figure 5:**
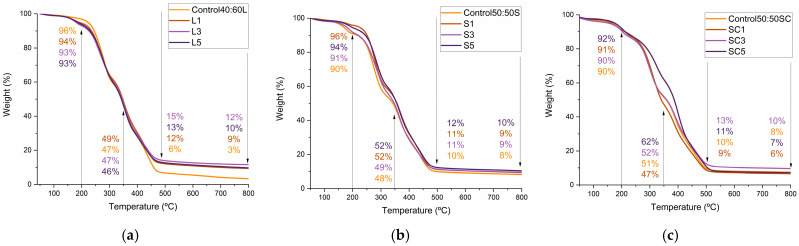
(**a**) TGA of lignin bio-polyol PU foams (free rise); (**b**) TGA of barley straw bio-polyol PU foams (free rise); (**c**) TGA of barley straw bio-polyol PU foams (closed mold).

The thermal stability of the polyurethane foams was evaluated by thermogravimetric analysis (Figure 5), with the aim of determining whether the incorporation of LCNF could mitigate polymer degradation and shift the maximum decomposition peaks toward higher temperatures, indicative of enhanced thermal resistance.

For free-rise samples, all LBP foams displayed comparable degradation profiles, with only minor differences relative to the control. Although the control foam showed slightly lower weight loss during the early stages of heating, its degradation became considerably more pronounced above 500 °C (Figure 5a). In contrast, LCNF reinforced foams retained up to four times more residual mass than the unfilled formulation (12% and 10% for L3 and L5, respectively, versus 3% for the unreinforced foam), demonstrating an enhanced resistance to thermal decomposition. A similar behavior was observed for the SBP foams (Figure 5b), although the improvement in final residue was insignificant (approximately 2% higher than the control), this result suggests a limited contribution of LCNF to thermal stabilization in this formulation.

In closed mold foams, synthesized from SBP, no improvement was observed with the increased addition of LCNF at the final analysis stages. Nevertheless, sample SC5 displayed a distinct enhancement in thermal stability between 250 and 450 °C, indicating that controlled expansion may influence heat transfer and network formation during curing (Figure 5c).

**Figure 6:**
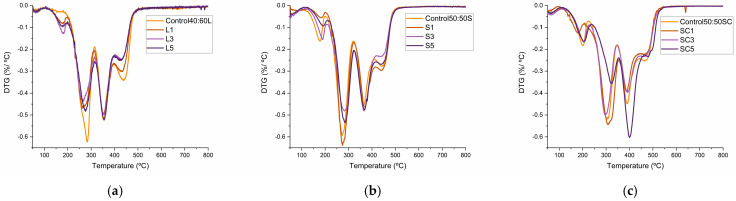
(**a**) DTG analysis of lignin bio-polyol PU foams (free rise); (**b**) DTG analysis of barley straw bio-polyol PU foams (free rise); (**c**) DTG analysis of barley straw bio-polyol PU foams (closed mold).

Derivative thermogravimetric (DTG) profiles revealed the typical multi-step decomposition behavior of polyurethane under nitrogen atmosphere (Figure 6). The first and second peaks (156–192 and 260–285 °C) correspond to the volatilization of residual components and the decomposition of unreacted bio-polyol and LCNF, respectively [41,56]. The third peak, appearing at ~350–370 °C, is associated with the degradation of hard-segment domains (urea, urethane and isocyanurate linkages) [57,58], while the final stage (420–440 °C) arises from the cleavage of C-C bonds and ester decomposition within the bio-polyol matrix [22].

Although LCNF addition did not significantly shift the maximum degradation temperatures, foams with SBP exhibited a consistent shift of 20–30 °C toward higher temperatures compared with their lignin-based counterparts, confirming the superior thermal robustness imparted by the multicomponent lignocellulosic feedstock (Figure 6a,b). Moreover, closed mold samples (Figure 6c) exhibited an additional shift of up to 40 °C, particularly in the SC5 sample, showing a delayed thermal decomposition. These findings reinforce the potential of LCNF-reinforced, straw-based polyurethane foams as potential thermally stable materials, suitable for applications where durability and fire resistance are critical performance criteria. Details about degradation peaks are shown in Table S3 (see Supplementary Material).

Additionally, the DSC thermograms revealed clear trends associated with both the bio-polyol source and the incorporation of LCNF. In general, foams produced with SBP exhibited glass transition temperatures (Tg) approximately 20 °C higher than those synthesized with LBP, reflecting the formation of a more efficiently crosslinked network. This result aligns with previous analysis, indicating that the lower steric hindrance and higher accessibility of hydroxyl groups in SBP increase segmental rigidity. Moreover, the incorporation of LCNF induced a moderate shift in Tg toward higher temperatures in both systems, consistent with the restricted polymer chain mobility caused by the formation of additional urethane linkages [27]. Interestingly, foams under closed-mold conditions showed lower Tg values; this decrease can be attributed to the morphological constraints imposed by the mold, which generate an anisotropic cell structure and lower effective crosslinking. Further details are shown in Table S4 of the Supplementary Material.

## 4. Conclusions

The valorization of barley straw allowed a systematic assessment of how each lignocellulosic component influences the structure-properties relationships of bio-based PU foams. The FTIR demonstrated that LCNF plays a dual role in the reaction mixture: a chemically active phase supplying additional hydroxyl groups that participate in urethane formation and a heterogeneous nucleating agent. The incorporation of LCNF markedly improved the mechanical performance of bio-based PU foams, with an optimal formulation observed at 5 wt.% in LBP foams and at 3 wt.% with the use of SBP, where compressive strength increased by up to 121% and 62.5%, respectively. Higher LCNF content in barley straw-derived foams led to slight declines, likely due to fiber agglomeration and saturation of the matrix with hydroxyl groups. LBP foams displayed a homogeneous appearance and low-density structure, suitable for flexible or comfort applications, whereas foams derived from whole-straw bio-polyol exhibited denser structures and higher load-bearing capacity, aligned with the requirements for structural materials. Thermogravimetric analysis confirmed enhanced stability in barley straw-derived foams, with degradation peaks shifting up to 30 °C, with respect to lignin bio-polyol foams.

Therefore, this study establishes a fully integrated, zero-waste valorization pathway for agricultural residues, offering a new framework to elucidate the specific role of each lignocellulosic fraction in the multifunctionality of complex polymer matrices such as polyurethane foams.

## Figures and Tables

**Table 1:** Polyurethane foam formulations.

Sample	Formulation (BP:CO) ^a^	LCNF (g)	LBP ^b^ (g)	SBP ^c^ (g)	CO (g)	Catalyst ^d^ (g)	Blowing Agent ^e^ (g)	Silicone Oil (g)	TDI (g)	R_NCO/OH_	Foaming Reaction
Control 50:50 L	50:50	0	10	0	10	0.4	0.4	0.4	11.5	0.66	No
Control 40:60 L	40:60	0	8	0	12	0.4	0.4	0.4	11.5	0.73	Yes
L1	40:60	0.33	8	0	12	0.4	0.4	0.4	11.5	0.73	Yes
L3	40:60	1.01	8	0	12	0.4	0.4	0.4	11.5	0.73	Yes
L5	40:60	1.71	8	0	12	0.4	0.4	0.4	11.5	0.73	Yes
L7	40:60	2.46	8	0	12	0.4	0.4	0.4	11.5	0.73	No
Control 60:40 S	60:40	0	0	12	8	0.4	0.4	0.4	11.5	0.65	No
Control 50:50 S	50:50	0	0	10	10	0.4	0.4	0.4	11.5	0.71	Yes
S1	50:50	0.33	0	10	10	0.4	0.4	0.4	11.5	0.71	Yes
S3	50:50	1.01	0	10	10	0.4	0.4	0.4	11.5	0.71	Yes
S5	50:50	1.71	0	10	10	0.4	0.4	0.4	11.5	0.71	Yes
S7	50:50	2.46	0	10	10	0.4	0.4	0.4	11.5	0.71	No
Control 60:40 SC	60:40	0	0	12	8	0.4	0.4	0.4	11.5	0.65	No
Control 50:50 SC	50:50	0	0	10	10	0.4	0.4	0.4	11.5	0.71	Yes
SC1	50:50	0.33	0	10	10	0.4	0.4	0.4	11.5	0.71	Yes
SC3	50:50	1.01	0	10	10	0.4	0.4	0.4	11.5	0.71	Yes
SC5	50:50	1.71	0	10	10	0.4	0.4	0.4	11.5	0.71	Yes
SC7	50:50	2.46	0	10	10	0.4	0.4	0.4	11.5	0.71	No

^a^ Bio-polyol: Castor oil. ^b^ Lignin bio-polyol. ^c^ Barley straw bio-polyol. ^d^ Dibutyltin dilaurate. ^e^ Water.

**Table 2:** Bio-polyols characterization.

		Lignin Bio-Polyol	Barley Straw Bio-Polyol
pH		2.80 ± 0.03	2.82 ± 0.05
Density (g/mL)		1.43 ± 0.07	1.23 ± 0.05
I_OH_ (mg KOH/g)		710.9 ± 9.4	625.7 ± 5.2
Acid number (mg KOH/g)		49.9 ± 8.3	22.8 ± 0.9
Viscosity (mPa·s)		6026.5 ± 12.0	6738.5 ± 156.3
Molecular weight	*M_W_* (g/mol)	21,348	25,436
	*M_N_* (g/mol)	20,554	23,996
	*PD*	1.04	1.06

**Table 3:** Quality index parameters.

	Parameters
Consistency (%)	0.93 ± 0.05
Nanosized fraction (%)	94.9 ± 6.4
Turbidity (NTU)	63.3 ± 2.6
Young’s Modulus (GPa)	9.3 ± 3.9
Macroscopic size (µm^2^)	9.4
Quality Index (%)	93.4

## Data Availability

The raw data supporting the conclusions of this article will be made available by the authors upon request.

## Supplementary Materials

The following supporting information can be downloaded at: https://www.mdpi.com/article/10.3390/polym17233142/s1. Figure S1: FTIR spectra of bio-polyols. Table S1: Mechanical characterization of PU foams. Table S2: Cell size values. Table S3: Temperature of decomposition peaks. Table S4: Glass transition temperatures of PU foams.

## Abbreviations

The following abbreviations are used in this manuscript:

LCNF	Lignin-containing cellulose nanofibers
PU	Polyurethane
CO	Castor oil
ASTM	American Society for Testing and Materials
TGA	Thermogravimetric analysis
TDI	Tolylene-2,4-diisocyanate
SEM	Scanning Electron Microscopy
