# Supplementary material for: A Dual Valorization Strategy of Barley Straw for the Development of High-Performance Bio-Based Polyurethane Foams
Source: Polymers (Basel). 2025 Nov 26;17(23):3142. doi: 10.3390/polym17233142 (PMC12694198; doi:10.3390/polym17233142)
Supplement: Supplementary file 1 [file polymers-17-03142-s001.zip › polymers-3987616-supplementary.pdf]

A Dual Valorization Strategy of Barley Straw for the Development of High-Performance Bio-based Polyurethane Foams

Marina Rodríguez[1,2], Esther Rincón[1,2], María Pinillos[1,2], Pablo E. Romero[3], Luis Serrano[1,2*]

1 Inorganic Chemistry and Chemical Engineering Department, BioPrEn RNM-940 Research Group, Universidad de Córdoba, Marie Curie Building (C-3), Ctra. Nnal. Km. 396, 14014 Córdoba, Spain.
2 Faculty of Science, Instituto Químico Para la Energía y el Medioambiente (IQUEMA), Universidad de Córdoba, Marie Curie Building (C-3), Ctra. Nnal. Km. 396, 14014 Córdoba, Spain.
3 Mechanical Engineering Department, Universidad de Córdoba, Leonardo da Vinci Building, Ctra. Nnal. Km. 396, 14014 Córdoba, Spain.
* Correspondence: iq3secal@uco.es

The FTIR spectra of the synthesized bio-polyols (Figure S1) exhibit characteristics bands confirming the successful liquefaction of lignin and lignocellulosic biomass, leading to the formation of hydroxyl-rich polyols. A broad band centered around 3300 cm^{-1}, corrrespond to stretching vibrations of hydroxyl groups (-OH). As shown in Figure S1, this band appears broader and more intense in SBP, indicating a higher abundance and diversity of hydroxyl functionalities derived from cellulose and hemicellulose structures. The band near 1725 cm^{-1} is attributed to the C=O stretching of ester, carbonyl or unconjugated ketones. Additionally, the peak at 1220 cm^{-1} assigned to C-O stretching of aryl ethers is more pronounced in LBP, reflecting its higher aromatic content and greater contribution from lignin-derived structural units [35].

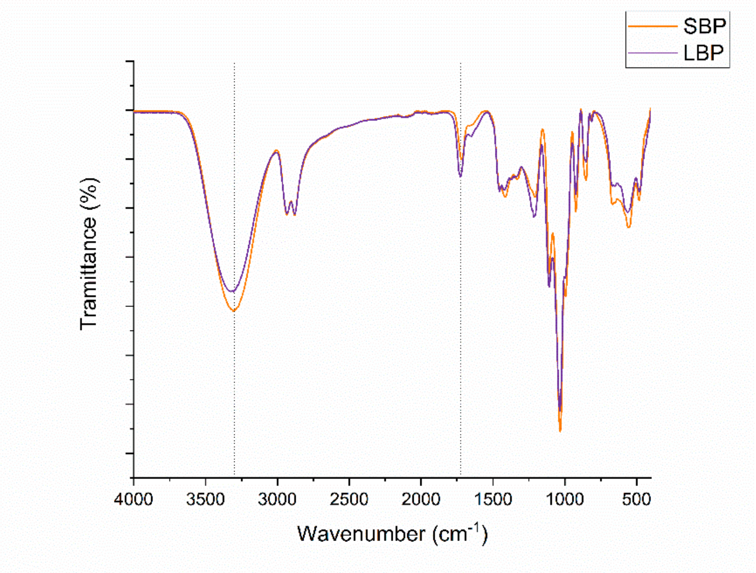

Figure S1. FTIR spectra of bio-polyols.

Table S1. Mechanical characterization of PU foams.

Sample	*Apparent density (kg/m^3)*	*Specific compressive strength (kPa· m^3/kg)*	*Specific Young's modulus (kPa· m^3/kg)*
Control40:60L	*125.78 ± 0.61*	*0.234 ± 0.015*	*3.35 ± 0.28*
L1	*54.07 ± 0.16*	*0.218 ± 0.006*	*2.85 ± 0.59*
L3	*134.30 ± 0.62*	*0.375 ± 0.015*	*4.78 ± 0.60*
L5	*218.90 ± 0.35*	*0.510 ± 0.042*	*7.07 ± 1.20*
Control50:50S	*213.46 ± 0.60*	*0.881 ± 0.176*	*19.90 ± 0.30*
S1	*292.42 ± 0.42*	*1.102 ± 0.189*	*20.30 ± 0.57*
S3	*324.34 ± 0.34*	*1.430 ± 0.148*	*26.00 ± 0.24*
S5	*195.28 ± 0.59*	*0.569 ± 0.206*	*9.14 ± 2.14*
Control50:50SC	*162.75 ± 0.37*	*0.725 ± 0.007*	*14.80 ± 3.50*
SC1	*195.72 ± 0.48*	*0.780 ± 0.086*	*15.03 ± 0.66*
SC3	*382.50 ± 1.70*	*0.980 ± 0.089*	*23.76 ± 4.35*
SC5	*219.20 ± 1.60*	*0.596 ± 0.180*	*8.86 ± 2.80*

Table S2. Cell size values.

**Sample**	**Average cell size (mm)**	**Cell wall tickness (μm)**
**Control40:60L**	0.619 ± 0.237	209.4 ± 112.6
**L1**	0.806 ± 0.123	220.5 ± 132.5
**L3**	0.480 ± 0.259	379.6 ± 129.7
**L5**	0.270 ± 0.131	412.2 ± 176.1
**Control50:50S**	1.034 ± 0.262	238.7 ± 114.4
**S1**	0.664 ± 0.208	325.3 ± 157.5
**S3**	0.598 ± 0.222	425.2 ± 207.9
**S5**	0.403 ± 0.167	424.8 ± 143.8
**Control50:50SC**	0.862 ± 0.246	301.6 ± 109.1
**SC1**	0.703 ± 0.149	277.1 ± 62.2
**SC3**	0.361 ± 0.245	327.9 ± 219.8
**SC5**	0.410 ± 0.184	480.2 ± 189.0

Table S3. Temperature of decomposition peaks.

Sample	*T_1 (℃)*	*T_2 (℃)*	*T_3 (℃)*	*T_4 (℃)*	*Final residue (800 ºC)*
Control40:60L	*156.02*	*283.84*	*354.89*	*439.56*	*3 %*
L1	*177.69*	*260.81*	*355.01*	*433.22*	*9 %*
L3	*182.95*	*267.94*	*354.42*	*429.88*	*12 %*
L5	*178.27*	*276.38*	*355.49*	*423.88*	*10 %*
Control50:50S	*178.05*	*273.22*	*364.98*	*443.26*	*8 %*
S1	*183.54*	*274.86*	*371.62*	*442.49*	*9 %*
S3	*188.81*	*282.38*	*366.48*	*443.77*	*9 %*
S5	*192.18*	*285.33*	*366.69*	*439.18*	*10 %*
Control50:50SC	*201.03*	*303.46*	*390.43*	*466.09*	*8 %*
SC1	*179.62*	*308.03*	*388.31*	*463.67*	*6 %*
SC3	*204.53*	*295.94*	*391.43*	*463.19*	*10 %*
SC5	*205.31*	*323.61*	*400.31*	*481.10*	*7 %*

Table S4. Glass transition temperatures of PU foams.

**Sample**	**Tg (˚C)**
**Control40:60L**	57.3
**L1**	55.2
**L3**	67.7
**L5**	69.6
**Control50:50S**	83.7
**S1**	86.4
**S3**	90.3
**S5**	67.1
**Control50:50SC**	72.0
**SC1**	84.1
**SC3**	75.0
**SC5**	70.5
